# [^18^F]FDG-PET accurately identifies pathological response early upon neoadjuvant immune checkpoint blockade in head and neck squamous cell carcinoma

**DOI:** 10.1007/s00259-021-05610-x

**Published:** 2021-12-27

**Authors:** Joris L. Vos, Charlotte L. Zuur, Laura A. Smit, Jan Paul de Boer, Abrahim Al-Mamgani, Michiel W. M. van den Brekel, John B. A. G. Haanen, Wouter V. Vogel

**Affiliations:** 1 Department of Head and Neck Oncology and Surgery, The Netherlands Cancer Institute, Amsterdam, The Netherlands; 2 Department of Maxillofacial Surgery, Amsterdam University Medical Center, Amsterdam, The Netherlands; 3 Department of Otorhinolaryngology and Head and Neck Surgery, Leiden University Medical Center, Leiden, The Netherlands; 4 Department of Pathology, The Netherlands Cancer Institute, Amsterdam, The Netherlands; 5 Department of Medical Oncology, The Netherlands Cancer Institute, Amsterdam, The Netherlands; 6 Department of Radiation Oncology, The Netherlands Cancer Institute, Plesmanlaan 121, 1066 CX Amsterdam, The Netherlands; 7 Department of Nuclear Medicine, The Netherlands Cancer Institute, Plesmanlaan 121, 1066 CX Amsterdam, The Netherlands

**Keywords:** Head and neck squamous cell carcinoma, Neoadjuvant immune checkpoint blockade, [^18^F]FDG-PET, Metabolic response assessment

## Abstract

**Purpose:**

To investigate the utility of [^18^F]FDG-PET as an imaging biomarker for pathological response early upon neoadjuvant immune checkpoint blockade (ICB) in patients with head and neck squamous cell carcinoma (HNSCC) before surgery.

**Methods:**

In the IMCISION trial (NCT03003637), 32 patients with stage II‒IVb HNSCC were treated with neoadjuvant nivolumab with (*n* = 26) or without (*n* = 6) ipilimumab (weeks 1 and 3) before surgery (week 5). [^18^F]FDG-PET/CT scans were acquired at baseline and shortly before surgery in 21 patients. Images were analysed for SUV_max_, SUV_mean_, metabolic tumour volume (MTV), and total lesion glycolysis (TLG). Major and partial pathological responses (MPR and PPR, respectively) to immunotherapy were identified based on the residual viable tumour in the resected primary tumour specimen (≤ 10% and 11–50%, respectively). Pathological response in lymph node metastases was assessed separately. Response for the 2 [^18^F]FDG-PET-analysable patients who did not undergo surgery was determined clinically and per MR-RECIST v.1.1. A patient with a primary tumour MPR, PPR, or primary tumour MR-RECIST-based response upon immunotherapy was called a responder.

**Results:**

Median ΔSUV_max_, ΔSUV_mean_, ΔMTV, and ΔTLG decreased in the 8 responders and were significantly lower compared to the 13 non-responders (*P* = 0.05, *P* = 0.002, *P* < 0.001, and *P* < 0.001). A ΔMTV or ΔTLG of at least − 12.5% detected a primary tumour response with 95% accuracy, compared to 86% for the EORTC criteria. None of the patients with a ΔTLG of − 12.5% or more at the primary tumour site developed a relapse (median FU 23.0 months since surgery). Lymph node metastases with a PPR or MPR (5 metastases in 3 patients) showed a significant decrease in SUV_max_ (median − 3.1, *P* = 0.04). However, a SUV_max_ increase (median + 2.1) was observed in 27 lymph nodes (in 11 patients), while only 13 lymph nodes (48%) contained metastases in the corresponding neck dissection specimen.

**Conclusions:**

Primary tumour response assessment using [^18^F]FDG-PET-based ΔMTV and ΔTLG accurately identifies pathological responses early upon neoadjuvant ICB in HNSCC, outperforming the EORTC criteria, although pseudoprogression is seen in neck lymph nodes. [^18^F]FDG-PET could, upon validation, select HNSCC patients for response-driven treatment adaptation in future trials.

**Trial registration:**

https://www.clinicaltrials.gov/, NCT03003637, December 28, 2016.

**Supplementary Information:**

The online version contains supplementary material available at 10.1007/s00259-021-05610-x.

## Introduction

Immune checkpoint blockade (ICB) of programmed cell death protein 1 (PD-1) leads to objective responses in 13–17% of patients with recurrent or metastatic head and neck squamous cell carcinoma (HNSCC) and significantly improves their overall survival compared to chemotherapy [1, 2]. Recent trials have shown that dual ICB of PD-1 and cytotoxic T-lymphocyte-associated protein 4 (CTLA-4) can be safely administered prior to definitive surgery and leads to pathologically confirmed responses in patients with various solid tumours [3–7]. In HNSCC, neoadjuvant combined anti-PD-1 and anti-CTLA-4 ICB lead to a major pathological response (MPR) in 20–35% of patients [6, 7]. Importantly, our group has recently demonstrated that none of the HNSCC patients with an MPR after neoadjuvant dual ICB has developed a tumour relapse, significantly superior to patients without an MPR [7]. While these results warrant validation, they could challenge the necessity of mutilating and functionally impairing surgery [8] and adjuvant (chemo)radiotherapy, and provide a rationale to investigate the feasibility of withholding or de-escalating standard-of-care in patients with a deep pathological response early upon neoadjuvant ICB.

Such a response-driven treatment adaptation requires a reliable biomarker to identify individual patients with a pathological response in the neoadjuvant time frame. With its widespread availability and established position in the clinic, imaging-based response evaluation is an attractive option. However, evaluation of CT and MR images according to the response evaluation criteria in solid tumours (RECIST [9]) has shown to underestimate the frequency and depth of pathological response after neoadjuvant ICB in various tumour types, including HNSCC [3, 4, 7, 10]. [^18^F]fluorodeoxyglucose(FDG)-PET-based metabolic response evaluation [11], on the other hand, has been shown to accurately identify pathological responses after two cycles of neoadjuvant ICB in patients with non-small cell lung cancer 3 to 5 weeks after start of treatment [12]. In addition, our group has recently demonstrated that HNSCC patients with an early primary tumour pathological response to neoadjuvant ICB are accompanied by a decrease in primary tumour total lesion glycolysis (TLG) assessed per [^18^F]FDG-PET in a 4-week timeframe [7]. Still, the exact value of [^18^F]FDG-PET as an imaging biomarker for early pathological response to neoadjuvant ICB in HNSCC remains unclear, as does its susceptibility to false-positivity (i.e. pseudoprogression) by ICB-induced immune activity in the primary tumour or involved or reactive lymph nodes [13]. Here, we report in detail on the [^18^F]FDG-PET scans acquired in the context of the IMCISION trial, wherein patients with locoregionally advanced HNSCC were treated with two cycles of nivolumab (anti-PD-1) monotherapy or nivolumab plus ipilimumab (anti-CTLA-4) before definitive surgery [7]. We aim to describe the manifestations of metabolic response, metabolic progression, and metabolic pseudoprogression after neoadjuvant ICB and explore [^18^F]FDG-PET scanning’s ability to predict pathological response early upon immunotherapy in patients with resectable HNSCC.

## Materials and methods

### Patients and trial interventions

IMCISION (NCT03003637) was an investigator-initiated, non-randomized, open-label phase Ib/IIa trial carried out at the Netherlands Cancer Institute (NKI), of which the methods and main results have been reported previously [7]. Briefly, adult patients with human papillomavirus (HPV)–related or HPV-unrelated, T2‒T4, N0‒N3b, resectable HNSCC of the oral cavity or oropharynx were eligible for inclusion. Patients with hypopharyngeal or laryngeal SCC were eligible too, but only 1 laryngeal HNSCC patient was accrued and had no scans available; this patient is not included in the current investigation. Patients with recurrent HNSCC were eligible if they were scheduled for curative surgery. All patients had a World Health Organization performance score of 0 or 1 and adequate bone marrow, liver, and kidney function. Critical exclusion criteria were distant metastases, a medical history of autoimmune disease, the use of immunosuppressive medication, or prior treatment with ICB targeting PD-1, PD-L1, or CTLA-4.

**Fig. 1:**
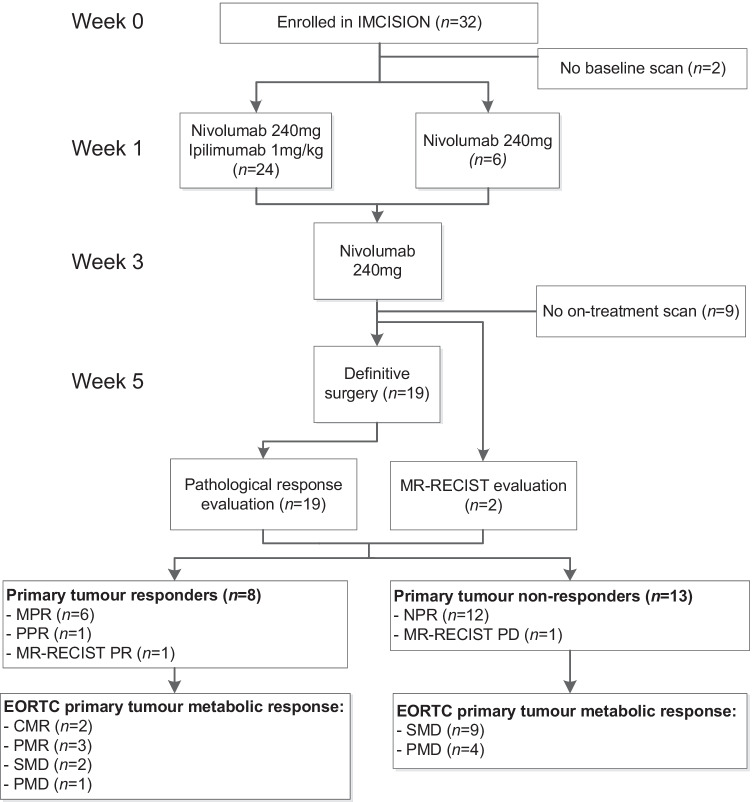
Flow chart of trial treatments, timelines, and patients included in IMCISION and their [^18^F]FDG-PET-based metabolic treatment response. Patients were treated with neoadjuvant nivolumab or nivolumab and ipilimumab (week 1), followed by nivolumab (week 3). Surgery was performed in week 5 or, ultimately, week 6. An evaluable baseline and on-treatment scan were obtained in 21 patients, of whom 6 had a major pathological response (MPR), 1 a partial pathological response (PPR), and 12 no pathological response (NPR) at their primary tumour site. Two patients did not undergo complete resection of their primary tumour and were classified according to MR-RECIST v.1.1, which was in agreement with physical examination in both cases: 1 RECIST-PR (responder) and 1 RECIST-PD (non-responder)

Patients underwent staging investigations at baseline (week 0), including tumour biopsy, laboratory investigations, MR imaging of the head and neck, ultrasound of the neck with fine-needle aspiration cytology, and total body [^18^F]FDG-PET. Staging was performed according to the 8th edition of the American Joint Committee on Cancer (AJCC) staging manual. Enrolled patients received 2 cycles of neoadjuvant ICB. Figure 1 details trial treatments and timelines. The first 6 patients were treated with nivolumab (240 mg flat dose) in weeks 1 and 3; the subsequent 26 patients received nivolumab (240 mg flat dose) and ipilimumab (1 mg/kg) in week 1, followed by nivolumab (240 mg flat dose) in week 3. On-treatment MR imaging and, if additional consent was given, [^18^F]FDG-PET were obtained at the end of week 4. Standard-of-care surgery was performed in week 5, or ultimately in week 6. Adjuvant (chemo)radiotherapy was performed if indicated according to institutional and national guidelines.

### Defining response to immunotherapy: pathological and MR-RECIST evaluation

The pathological response of the primary tumour was determined by a head and neck pathologist (LS) on H&E-stained sections of the resected specimen. The proportion of viable tumour cells within the histologically identifiable tumour bed was quantified as a percentage and compared to the percentage of viable tumour cells within the baseline biopsy to compensate for a low baseline viable tumour cell count. The degree of pathological tumour regression was determined by calculating the percentage change in the primary tumour viable tumour cell percentage from baseline biopsy to the on-treatment resected specimen. Patients with ≤ 10% residual viable tumour cells and 90‒100% tumour regression at the primary tumour site at time of surgery had a major pathological response (MPR). Patients with ≤ 50% residual viable tumour cells and 50‒89% regression had a partial pathological response (PPR), and patients with any percentage of residual viable tumour cells but < 50% regression had no pathological response (NPR) [14]. Two patients did not undergo curative surgery and were thus unevaluable for pathological efficacy. For the present analyses, these patients were classified according to their MR-RECIST v.1.1 response on the on-treatment scan compared to the baseline scan.

To facilitate pathological correlation of [^18^F]FDG-PET-identifiable lymph nodes after ICB, the head and neck surgeon marked the different cervical nodal levels during surgery using beads of different colours, according to our institutional neck dissection protocol. Lymph nodes were evaluated by LS. If a metastasis was present, ICB response was determined using the same cut-offs for pathological response at the primary tumour site (MPR, PPR, or NPR).

Overall, a patient with a primary tumour MPR, PPR, or, in the absence of pathological response evaluation, MR-RECIST-based response upon immunotherapy was called a responder. Having a (pathological) response in one or more lymph nodes in the absence of at least a partial response at the primary tumour site was not sufficient to be classified as a responder.

### [^18^F]FDG-PET image acquisition

PET-scans were obtained at baseline and, if the patient consented, in the week prior to surgery using a Gemini TF, TF Big bore, or Vereos PET/CT scanner (Philips, Cleveland). One patient underwent baseline scanning in the referring hospital on a Biograph M20 (Siemens, Munich). Patients were instructed to fast for at least 6 h before the scan. If the blood glucose level did not exceed 12 mmol/L, the patient received 190‒280 MBq [^18^F]FDG (according to BMI) intravenously. Sixty minutes later, 3D PET images were obtained with 3 min per bed position for the head-neck area and 2 min per bed position for the neck-thighs. For anatomical correlation, low dose CT was acquired with parameters including 120 kV, 40 mAs with dose optimization, and slice interval and thickness 2 mm. All image sets from all scanners used were acquired and reconstructed according to EARL specifications to allow standardized quantification.

### PET image analysis

The [^18^F]FDG-PET images were evaluated jointly by two researchers (JV and WV), one of whom is a head and neck nuclear physician (WV), using Osirix software v11.0.1 (Pixmeo, Switzerland). A spherical volume of interest containing the whole area of [^18^F]FDG-activity was manually grown around the primary tumour, in which SUV_max_ was determined. Next, SUV_mean_ (the mean SUV of voxels within the volume of interest) was calculated in the subvolume with an intensity ≥ 50% of SUV_max_, as is the clinical standard in our institute. This volume also defined the metabolic tumour volume (MTV). Total lesion glycolysis (TLG) was calculated by multiplying MTV with SUV_mean_. For the on-treatment scan, 50% SUV_max_ of the baseline scan was used to calculate MTV and TLG. SUV_mean_, MTV, and TLG could not be reliably calculated if the primary tumour could not be clearly visualized or accurately distinguished from background [^18^F]FDG-uptake. As determined at the immunotherapy symposium of the European Association of Nuclear Medicine 2017 annual meeting [13], both the PERCIST or the European Organization for Research and Treatment of Cancer (EORTC) PET study group’s response criteria may be used in the assessment of immunotherapy response. According to routine clinical practice at our institute, we assessed response using the EORTC recommendations [15]: complete metabolic response (CMR) was defined as a complete resolution of FDG uptake in the primary tumour from baseline to on-treatment, partial metabolic response (PMR) as a > 25% decrease in primary tumour SUV_max_ from baseline to on-treatment, and progressive metabolic disease (PMD) as a > 25% increase in primary tumour SUV_max_ from baseline to on-treatment. As we determined pathological response in the primary tumour separately from the response in the lymph nodes, the appearance of new [^18^F]FDG-avid lesions was not classified as PMD. All patients not meeting the criteria for CMR, PMR, or PMD were classified as having stable metabolic disease (SMD).

In case a lymph node, with or without metastasis, showed notable metabolic activity on the baseline or on-treatment scan (or both), SUV_max_, SUV_mean_, MTV, and TLG were determined. In case of metabolic activity on only the baseline or on-treatment scan, background metabolism in the same node on the other scan was measured for reference. All lymph nodes detected by [^18^F]FDG-PET prior to treatment were clinically diagnosed by ultrasound and, if needed, fine needle aspiration cytology. If an [^18^F]FDG-PET-identifiable lymph node resided in a level where at least one node was pathologically tumour-positive, that avid lymph node was assumed to be tumour-positive. A lymph node detected by [^18^F]FDG-PET was considered tumour-negative only if all dissected nodes in that particular level were pathologically tumour-negative. We defined a lymph node as pseudoprogressive if there was an increase in SUV_max_ from baseline to on-treatment in the absence of HNSCC metastasis upon pathological examination.

### Statistical considerations

All statistics were descriptive. SUV_max_, SUV_mean_, MTV, and TLG values at baseline and on-treatment and the (percent) change between the two time points are reported as medians with their interquartile range (IQR). Median values are compared between responders and non-responders using a Wilcoxon rank-sum test. Within the same patient, baseline and on-treatment values were compared using a Wilcoxon signed-rank test. Time to progression (TTP) was defined as the time from surgery to the first local, regional, or distant HNSCC relapse. The 2 patients that did not undergo surgery were thus excluded from TTP analysis. Overall survival (OS) was defined as the time between the first ICB dose and death from any cause (i.e. including the 2 patients that did not undergo surgery). Survival estimates were made using the Kaplan–Meier method; responders and non-responders are compared using a log-rank test. Median follow-up time was calculated using the inverse Kaplan–Meier method. The performance of a PET parameter as a diagnostic test for detecting response was assessed per receiver operating characteristic (ROC) and the area under the ROC curve. All tests were two-sided, and a *P*-value < 0.05 was considered statistically significant. All statistical analyses were performed in SPSS Statistics version 25.0 (IBM Corp, Armonk, NY, USA) and GraphPad Prism version 9.0.0 (GraphPad Software, San Diego, CA, USA).

## Results

### Patient characteristics, pathologic and metabolic treatment response

[^18^F]FDG-PET scans were obtained at baseline and a median of 24 (IQR 3) days after the start of ICB in 21 of 32 IMCISION patients. Thirteen patients underwent imaging on the same scanner at both time points. Different scanners from the same manufacturer were used in 7 patients, while 1 patient was scanned on an EARL-calibrated machine from another manufacturer in the referring hospital. Definitive surgery was performed in 19 of these 21 patients, a median of 3 days (IQR 0) after the on-treatment scan: 2 patients were ineligible for surgery due to progressive disease or synchronous incurable oesophageal carcinoma.

Twenty of 21 PET-analysable patients had HPV-unrelated HNSCC, and 18 had an oral cavity carcinoma. Six patients (of whom 5 non-responders) had recurrent disease after previous concurrent cisplatin- or cetuximab-radiotherapy (3 patients), surgery with postoperative radiotherapy (1 patient), or surgery only (2 patients). Detailed baseline and neoadjuvant treatment characteristics are shown in Table 1.

**Table 1:** Baseline characteristics of [^18^F]FDG-PET-evaluable patients enrolled in the IMCISION trial. Patients with a major or partial pathological response to neoadjuvant ICB at the primary tumour site, and the patient without pathological response evaluation but with a response based on MR-RECIST v.1.1, were defined as ‘responders’. Patients without a pathological response or, in the absence of pathological evaluation, an MR-RECIST response at the primary tumour site were defined as ‘non-responders’. Tumours were staged according to the American Joint Committee on Cancer’s staging manual (8th edition)

	Total (*n* = 21)	Responders (*n* = 8)	Non-responders (*n* = 13)
Age, median (range)	66 (22–78)	59 (51–76)	66 (22–78)
Sex, *n* (%)			
Male	12 (57)	6 (75)	6 (46)
Female	9 (43)	2 (25)	7 (54)
Tumour site, *n* (%)			
Oral cavity	18 (86)	7 (88)	11 (85)
Oropharynx	3 (14)	1 (13)	2 (15)
Tumour HPV status, *n* (%)			
Positive	1 (5)	1 (13)	0
Negative	20 (95)	7 (88)	13 (100)
cT-stage, *n* (%)			
2	3 (14)	3 (38)	0
3	9 (43)	2 (25)	7 (54)
4	9 (43)	3 (38)	6 (46)
cN-stage, *n* (%)			
0	12 (57)	4 (50)	8 (62)
1	5 (24)	3 (38)	2 (15)
2	4 (19)	1 (13)	3 (23)
Clinical disease stage, *n* (%)			
II	2 (10)	2 (25)	0
III	6 (29)	3 (38)	3 (23)
IV	7 (33)	2 (25)	5 (39)
Recurrent	6 (29)	1 (13)	5 (39)
Neoadjuvant regimen			
Nivolumab	3 (14)	0	3 (23)
Nivolumab + ipilimumab	18 (86)	8 (100)	10 (77)

Seven of 21 patients had a pathological response at their primary tumour site, including 6 patients with an MPR and one with PPR. Twelve patients had no pathological response (Fig. 1). Of the two patients who did not undergo surgery, one had apparent clinical primary tumour regression and a partial response based on MR-RECIST v.1.1 (grouped with the pathological responders), and one had biopsy-proven MR-RECIST progressive disease (grouped with the pathological non-responders). In all, 8 patients (6 MPR, 1 PPR, 1 MR-RECIST-based response) were responders, and 13 patients (12 NPR, 1 clinical non-response) were non-responders (Fig. 1).

### EORTC metabolic response assessment underestimates incidence and depth of primary tumour pathological response early upon neoadjuvant immunotherapy

**Fig. 2:**
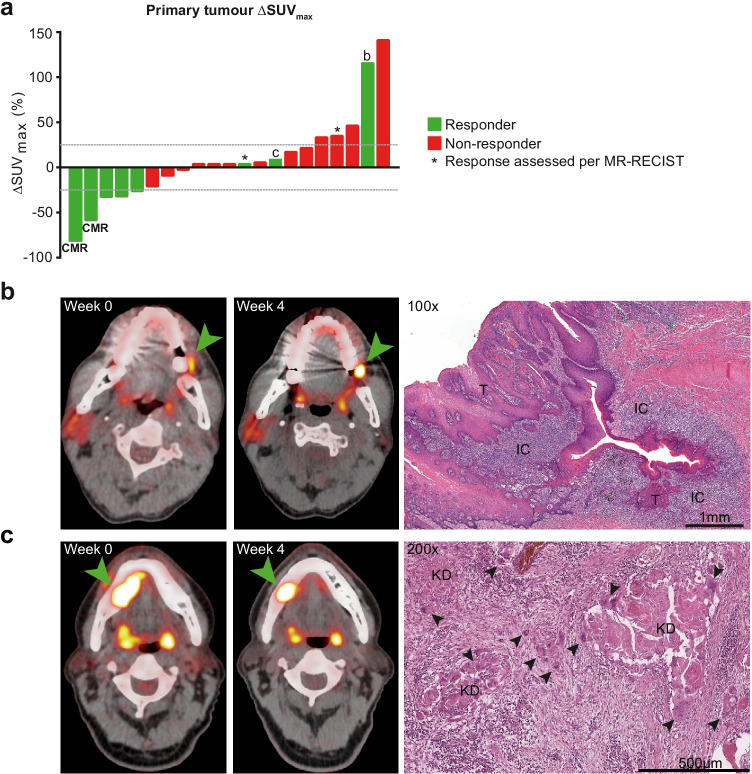
SUV_max_ waterfall plot and two cases illustrating the EORTC criteria’s relative inaccuracy to determine pathologic response at the primary tumour site early upon neoadjuvant ICB in HNSCC. **a** Waterfall plot presenting the percentage change in primary tumour SUV_max_ from baseline to on-treatment per individual patient. Green bars represent pathological responders, red bars non-responders. Patients in whom response was assessed per MR-RECIST are marked with an asterisk. Dotted grey lines at 25% and − 25% represent the EORTC criteria for progressive metabolic disease (PMD) and partial metabolic response (PMR), respectively; patients in between both lines had stable metabolic disease SMD. Two patients visually had a complete metabolic response (marked CMR), though the SUV_max_ did not become 0. Patients marked with **b** and **c** are further illustrated under **b** and **c**, respectively. **b** A patient with clinically rT2N0 carcinoma of the left cheek mucosa demonstrated primary tumour PMD after 2 cycles of nivolumab + ipilimumab, with a SUV_max_ increase from 5.4 to 11.7 (117%). Evaluation of the surgically resected specimen (right panel) revealed a major (near-complete) pathological response, with some viable residual tumour (‘T’) surrounded by a dense infiltrate of immune cells (‘IC’). **c** A patient with cT4aN2b HNSCC of the floor of the mouth shows a SUV_max_ increase from 15.9 to 17.5 (10%). Correlative histopathology shows a partial pathological response: 69% of the histologically identifiable tumour bed is taken up by keratinous debris (KD) under apparent clearance of multinucleated giant cells and foamy macrophages (arrows)

Five of the 8 responders had a CMR (2) or PMR (3) after neoadjuvant ICB according to EORTC criteria. Two responding patients had SMD and 1 had PMD (Fig. 1). A waterfall plot illustrating individual patients’ ΔSUV_max_ is shown in Fig. 2a. The responder marked with *b* in Fig. 2a had a SUV_max_ increase of 117%, yet the surgical specimen revealed an MPR with 94% cancer cell regression surrounded by a dense population of infiltrating immune cells (Fig. 2b). The two responders with SMD, one of whom had a PPR and is marked with *c* in Fig. 2a, demonstrated a decrease in the volume of metabolic activity at the primary tumour site in the absence of a SUV_max_ decrease (Fig. 2c).

### Volumetric [^18^F]FDG-PET metabolic primary tumour response assessment accurately identifies patients responsive to neoadjuvant ICB and favourable survival

**Fig. 3:**
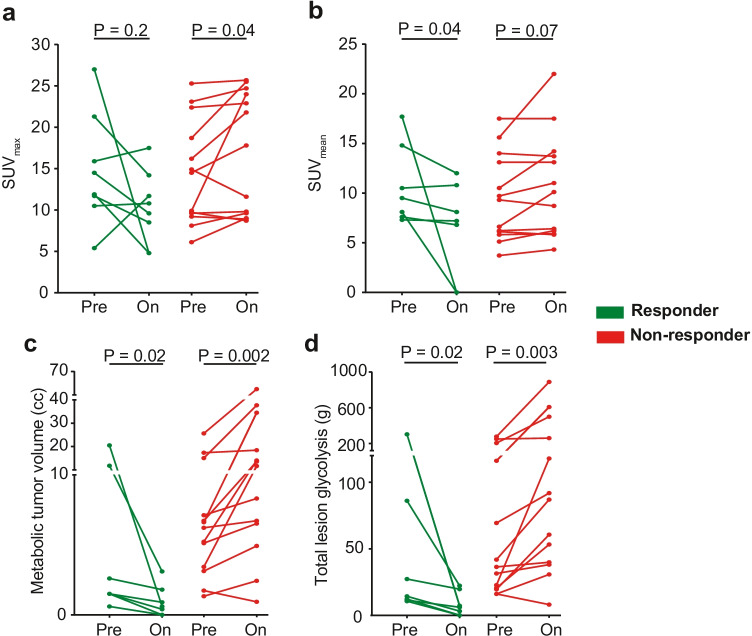
Individual primary tumour [^18^F]FDG-PET-based metabolic parameters at baseline (Pre) and on-treatment (On). **a**‒**d** Change in primary tumour SUV_max_ (**a**), SUV_mean_ (**b**), metabolic tumour volume (MTV, **c**), and total lesion glycolysis (TLG, **d**) from baseline to on-treatment. Patients with a response at their primary tumour site are shown in green, and patients without a primary tumour ICB response are shown in red. The 2 patients with a complete metabolic response are included with values ‘0’ for on-treatment SUV_mean_, MTV, and TLG. *P*-values were calculated using a Wilcoxon signed-rank test. Primary tumour SUV_mean_, MTV, and TLG could not be determined in the patient who had primary tumour metabolic pseudoprogression based on SUV_max_ (illustrated in Fig. 2b); this patient was excluded from **b**‒**d**. Please note that *y*-axes of **c** and **d** were interrupted to facilitate visualization

The median baseline and on-treatment primary tumour SUV_max_, SUV_mean_, MTV, and TLG and their percentage change from baseline to on-treatment are shown in Table 2. The medians of all parameters decreased from baseline to on-treatment in the responders’ group, whereas they increased in non-responding patients. The most profound change was observed in MTV and TLG, with a median of − 74 and − 77% for responding patients, and + 85% and + 108% for non-responding patients, respectively. In a paired analysis of baseline and on-treatment scans of individual patients, SUV_max_ did not change significantly in the responders’ group (*P* = 0.2). In contrast, SUV_mean_ (*P* = 0.04) and MTV and TLG (both *P* = 0.02) decreased significantly in all patients in the responders’ group (Fig. 3).

**Table 2:** Primary tumour [^18^F]FDG-PET-parameters at baseline and on-treatment and their percentage change in primary tumour responders and non-responders. *P*-values are calculated using a Wilcoxon rank-sum test. *IQR*, interquartile range; *SUV*, standardized uptake value; *MTV*, metabolic tumour volume; *TLG*, total lesion glycolysis

Metabolic parameter	Responders (*n* = 8)	Non-responders (*n* = 13)	*P*-value
Baseline, median (IQR)			
SUV_max_	14.5 (9.6)	14.5 (11.1)	0.8
SUV_mean_	9.5 (7.2)	9.3 (7.6)	0.3
MTV	1.5 (10.3)	6.2 (7.8)	0.2
TLG	14.3 (74.7)	36.0 (136.3)	0.2
On-treatment, median (IQR)			
SUV_max_	9.6 (9.4)	17.8 (15.1)	0.06
SUV_mean_	7.2 (10.8)	10.1 (7.9)	0.2
MTV	0.6 (1.8)	11.6 (20.8)	< 0.001
TLG	6.1 (19.7)	86.4 (334.4)	< 0.001
%Δ, median (IQR)			
%ΔSUV_max_	− 33.3 (62.6)	6.9 (36.4)	0.05
%ΔSUV_mean_	− 14.7 (98.6)	3.2 (29.5)	0.002
%ΔMTV	− 73.7 (60.0)	84.6 (147.8)	< 0.001
%ΔTLG	− 77.3 (53.7)	108.0 (206.0)	< 0.001

Patients in the non-responding group had a significant increase in SUV_max_ (*P* = 0.04), MTV (*P* = 0.002), and TLG (*P* = 0.002, Fig. 3). One patient without a primary tumour pathological response, however, showed a decrease in SUV_max_ (− 22%), SUV_mean_ (− 7%), MTV (− 47%), and TLG (− 51%, Supplementary Fig. 1). While not meeting pathological response criteria, this patient did have 22% pathological primary tumour regression and a major pathological response in the largest lymph node metastasis.

**Fig. 4:**
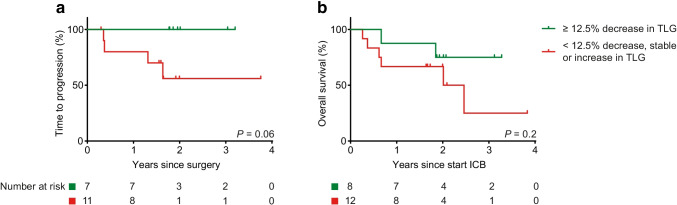
Kaplan–Meier survival estimates of patients with or without a total lesion glycolysis (TLG)–based primary tumour metabolic response. **a** Time to progression (TTP) since surgery of patients with a ≥ 12.5% decrease in TLG at primary tumour site (green) from baseline to on-treatment and patients without a ≥ 12.5% decrease (red). Only patients who underwent surgery are included here. **b** Overall survival since the start of ICB, for the same TLG-based metabolic response groups. The 2 patients who did not undergo surgery are included here. The deceased patients with a TLG-based metabolic response all died disease-free of causes unrelated to HNSCC. *P*-values were calculated using a log-rank test

The percent change in MTV and TLG as diagnostic tests for a pathological or MR-RECIST-based response early upon immunotherapy outperformed the EORTC criteria in terms of accuracy (Table 3). Using ΔTLG of − 12.5% as a threshold, patients with a TLG-based metabolic response at the primary tumour site who underwent surgery had a superior TTP compared to patients without a TLG-based metabolic response (Fig. 4a) at a median follow-up of 23.0 months since surgery (log-rank *P* = 0.06). OS since the start of ICB did not differ between the ΔTLG groups (log-rank *P* = 0.3, Fig. 4b). Of note, SUV_mean_, MTV, and TLG of the tumour of the patient with an MPR and a 117% increase in SUV_max_ (shown in Fig. 2b) could not be accurately calculated from the baseline scan due to poor distinction from surrounding normal tissue avidity, and were thus excluded. These data indicate that [^18^F]FDG-PET and particularly MTV and TLG may be accurate and early surrogate biomarkers for primary tumour ICB response and favourable TTP upon neoadjuvant immunotherapy prior to extensive surgery in HNSCC.

**Table 3:** Receiver operating characteristic describing the value of the percentage change in different FDG-PET parameters at the primary tumour site as surrogate marker for a pathological response early upon neoadjuvant ICB. *AUC*, area under the curve; *SUV*, standardized uptake value; *MTV*, metabolic tumour volume; *TLG*, total lesion glycolysis

Metabolic parameter	AUC	Threshold	Sensitivity	Specificity	Accuracy
%ΔSUV_max_	0.760	− 25%	1	0.63	0.86
%ΔSUV_mean_	0.901	− 8.5%	1	0.71	0.90
%ΔMTV	0.978	− 12.5%	0.92	1	0.95
%ΔTLG	0.978	− 12.5%	0.92	1	0.95

### Cervical lymph node metabolic response assessment is troubled by pseudoprogression

Pathological assessment of the cervical lymph nodes was performed in the 19 patients undergoing surgery (7 primary tumour responders, 12 non-responders). As reported previously [7], response to neoadjuvant ICB at the lymph node metastatic sites was not always congruent with the ICB-response at the primary tumour site. In all, only 5 of the 33 (15%) pathologically tumour-positive lymph nodes shared among 3 patients (of whom 1 with primary tumour MPR and 2 primary tumour NPR) showed evidence of PPR or MPR.

**Fig. 5:**
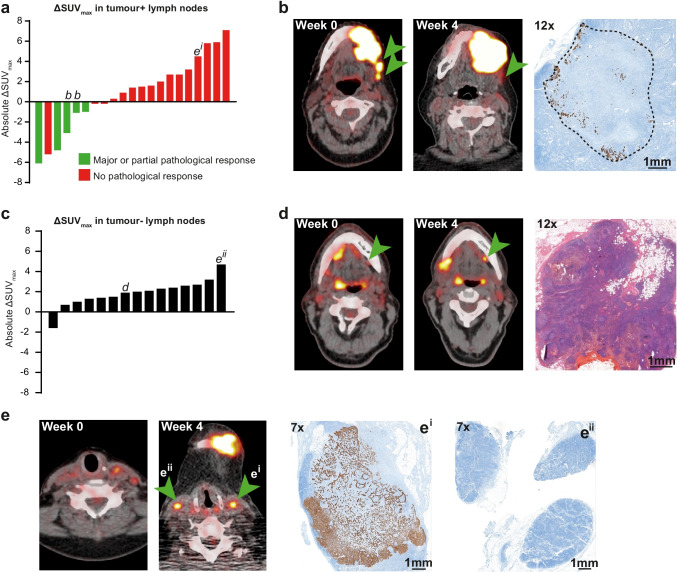
Metabolic responses, progression, and pseudoprogression in cervical lymph nodes. **a** Waterfall plot showing the absolute change in SUV_max_ from baseline to on-treatment for pathologically proven lymph node metastases in the neck dissection specimens. Bar colour indicates ICB response (green) or non-response (red). Bars marked **b** and **e**^**i**^ are further detailed under **b** and **e**^**i**^, respectively. **b** A patient with cT4aN2c HNSCC of the left alveolar process of the mandible showed PMD at the primary tumour site (SUV_max_ + 35%, SUV_mean_ + 35%, MTV + 144%, and TLG + 230%). Two ipsilateral level 2 lymph nodes (arrows) showed a SUV_max_ decrease from 8.8 to 5.7 (− 35%) and 6.6 to 5.6 (− 15%). Correlative keratin 14-stained pathology slides revealed one node with disturbed architecture but little viable tumour (12 × image), corresponding to an MPR. The other level 2 lymph node showed a PPR (not shown). **c** Waterfall plot showing the absolute change in SUV_max_ from baseline to on-treatment for pathologically proven tumour-negative lymph nodes. Bars marked with **d** and **e**^**ii**^ are further detailed under **d** and **e**^**ii**^, respectively. **d** A patient with a SUV_max_ increase from 3.4 to 5.3 (56%) in a left (contralateral) level 1b lymph node after neoadjuvant ICB (arrows). Correlative H&E slide of the left level 1b neck dissection specimen revealed no lymph node metastases. This patient’s primary tumour showed a partial pathological response (shown in Fig. 2c). **e** Level 3 transversal [^18^F]FDG-PET and keratin 14-stained pathology images of the same patient shown under **b**. Two level 3 nodes are detected: one left (ipsilateral, marked **e**^**i**^) with an SUV_max_ increase from 4.0 to 8.5, and one right (contralateral, marked **e**^**ii**^) with an SUV_max_ increase from 4.1 to 8.8. Correlative keratin 14-stained pathology slides showed a metastasis in level 3 left without evidence of ICB response (**e**^**i**^), while none of the resected right level 3 nodes contained tumour (**e**^**ii**^)

Twenty-one of 33 pathologically confirmed lymph node metastases could be reliably identified on [^18^F]FDG-PET (Fig. 5a). The 5 metastases with a PPR or MPR (example in Fig. 5b) showed a significant decrease in SUV_max_ (median 3.1, Wilcoxon signed rank *P* = 0.04). Three other lymph node metastases had a decrease in SUV_max_ in the absence of a pathological response (− 0.2, − 0.2, and − 5.2); the metastasis with a − 5.2 SUV_max_ decrease showed evidence of an ICB response, though not sufficient for a PPR. Thirteen lymph node metastases had an increase in SUV_max_ (median + 2.7, Wilcoxon signed rank *P* = 0.001), all without a pathological ICB response.

Fifteen lymph nodes in 7 patients that were suspected of harbouring HNSCC metastases based on [^18^F]FDG-PET turned out to be pathologically tumour-negative (Fig. 5c). Fourteen showed an increase in SUV_max_ from baseline to on-treatment (median + 2.1, Wilcoxon signed rank *P* = 0.002) and were considered pseudoprogressive (example in Fig. 5d). In one patient, a 1.9 increase in SUV_max_ in a contralateral lymph node led to escalation of surgery to include a bilateral neck dissection: histopathology revealed no contralateral metastases (Fig. 5d).

Median baseline and on-treatment SUV_max_ and their difference (absolute and per cent) for all 27 lymph nodes with a SUV_max_ increase after neoadjuvant ICB (13 tumour-positive and 14 tumour-negative) are shown in Table 4. The on-treatment SUV_max_ of the 13 tumour-positive nodes (7.2, IQR 4.1) was significantly higher than of the 14 tumour-negative nodes (4.8, IQR 1.1, *P* = 0.02). Still, distinguishing between true- and pseudoprogression in the cervical lymph nodes on [^18^F]FDG-PET is problematic, not least because these phenomena may be present simultaneously within the same patient and irrespective of the ICB responses in other lymph node metastases and the primary tumour, as illustrated in Fig. 5e.

**Table 4:** SUV_max_ at baseline and on-treatment and their percentage change of all 27 lymph nodes with a SUV_max_ increase, stratified per the presence of metastases in the pathology report. *P*-values are calculated using a Wilcoxon rank-sum test. *SUV*, standardized uptake value

Metabolic parameter, median (IQR)	Tumour-positive lymph nodes (*n* = 13)	Tumour-negative lymph nodes (*n* = 14)	*P*-value
Baseline SUV_max_	4.0 (4.4)	2.9 (1.4)	0.2
On-treatment SUV_max_	7.2 (4.1)	4.8 (1.1)	0.02
Absolute ΔSUV_max_	2.7 (3.7)	2.1 (1.3)	0.9
%ΔSUV_max_	67.8 (63.0)	69.9 (76.8)	0.4

Due to insufficiently avid and bulky disease at the metastatic sites, MTV and TLG could only be determined at baseline and on-treatment in 2 lymph node metastases with a response (of which one is shown in Fig. 3f) and greatly decreased in both nodes (MTV: − 99 and − 85%, TLG: − 99 and − 88%). MTV and TLG increased from baseline to on-treatment in the 2 non-responsive metastases for which they could be calculated (MTV: + 167 and + 533%, TLG: + 178 and + 748%).

## Discussion

Immune checkpoint blockade has become standard of care for patients with recurrent or metastatic HNSCC, and recent trial data demonstrate that ICB may be safely and effectively integrated into curative treatment as neoadjuvant therapy [6, 7, 16]. The relatively low major pathological response rate after neoadjuvant dual ICB with anti-PD-1 and anti-CTLA-4 of 20–35% in HNSCC, however, underlines the need to select of patients likely to respond [6, 7]. While several pre-treatment biomarkers, reviewed in [17], have been proposed, only the tumour PD-L1 combined positive score has entered clinical practice in the R/M HNSCC setting [2]. In the absence of reliable predictive biomarkers prior to treatment, on-treatment biomarkers identifying individual patients with a clinically relevant response early upon ICB may guide decision-making in future clinical trials investigating response-driven treatment adaptation. Our research suggests that MTV and TLG based on [^18^F]FDG-PET are promising surrogate biomarkers for primary tumour pathologic response and favourable disease-specific clinical outcome after neoadjuvant ICB in HNSCC and could, upon validation in an independent series, select patients for response-driven treatment adaptation in future trials.

Treatment response assessment per RECIST-criteria [9] based on CT or MR imaging has long been the gold standard for objectifying ICB response in a palliative setting and is a widely reported endpoint in clinical trials. For [^18^F]FDG-PET-based response evaluation, the EORTC [15] and PERCIST [11] criteria have been formulated, where metabolic response is determined based on a decrease in SUV_max_ or, respectively, SUL_peak_. ICB’s mechanism of action, recruiting host immune cells to infiltrate and clear a tumour, may result in a lesion to remain metabolically stable or even progress while it is, in fact, responding to treatment. To overcome pseudoprogression, the iRECIST criteria [18] for CT or MRI and iPERCIST [19] criteria for [^18^F]FDG-PET were developed, where an additional scan at a later time point is required to confirm or refute actual progressive disease. However, the neoadjuvant time frame does not offer the months needed to perform a reliable first RECIST or EORTC/PERCIST-based response assessment, let alone an additional confirmatory scan necessary per iRECIST or iPERCIST. Consequently, objective response rates assessed per RECIST have been shown to underestimate both the depth and incidence of pathological responses to neoadjuvant ICB in melanoma, colon cancer, non-small cell lung cancer, and HNSCC [3, 4, 6, 7, 10]. Therefore, unidirectional RECIST tumour measurements performed on CT or MR imaging seem unsuitable to predict an early pathological response upon ICB treatment accurately.

We have herein shown that while [^18^F]FDG-PET response assessment per EORTC criteria identifies some responders, it still yields an underestimation of the pathological response and results in pseudostable or pseudoprogressive disease at the primary tumour site in 3 of 8 HNSCC patients (38%) in IMCISION. In a trial investigating neoadjuvant sintilimab (anti-PD-1) in patients with resectable non-small cell lung cancer, Tao et al. noted that one patient with a PPR after neoadjuvant sintilimab (anti-PD-1) was classified as having PMD per PERCIST, while MTV and TLG did decrease with 60 and 50%, respectively [12]. Similarly, two other reports on metabolic ICB response assessment in patients with metastatic non-small cell lung cancer treated with nivolumab have shown that a decrease in TLG outperforms SUV_max_ when used as an early (2‒4 weeks after therapy initiation) biomarker for efficacy and progression-free survival [20, 21]. We herein propose that a decrease in primary tumour MTV and TLG accurately predicts primary tumour pathological response 4 weeks after start of neoadjuvant ICB in HNSCC patients. Importantly, we further show that none of the patients with a decrease in primary tumour MTV or TLG has developed a tumour relapse after 23 months postsurgical follow-up, superior to HNSCC patients without an MTV- or TLG-based metabolic response, and irrespective of the presence or absence of pathological response in these patients’ lymph node metastases.

Using MTV and TLG as biomarkers for ICB response early on-treatment has limitations. First, accurate computation of MTV and TLG requires a tumour bulk that can be accurately demarcated from [^18^F]FDG-avidity in the surrounding tissue. While this is in general not a problem in the locally advanced HNSCC setting, one patient in the present trial had a barely avid T2 tumour of the cheek mucosa, of which MTV and TLG could not be calculated at baseline. Similarly, metastatic HNSCC in cervical lymph nodes is often not sufficiently bulky and avid. Second, while MTV and TLG are more accurate than SUV_max_, they too are most likely not free from false-negativity through immune-induced pseudostable or -progressive disease, as has been shown in non-small cell lung cancer [21]. While we were unable to provide quantitative evidence in this research, the patient in whom baseline MTV and TLG were incalculable showed visually evident metabolic progression after treatment yet had a major pathological response at the primary tumour site. From a practical point of view, finally, adherence to an intensive protocol encompassing ICB and repeated metabolic response assessment in the short neoadjuvant time frame may be challenging for some patients with advanced HNSCC, a patient population characterized by alcohol and tobacco abuse and a low socio-economic status [22, 23].

Metabolic cervical lymph nodal pseudoprogression, herein defined as the increase of nodal avidity after neoadjuvant ICB in the absence of tumour, was seen in 14 of the 27 evaluable nodes (52%) in the present trial. Schoenfeld et al. reported that cervical lymph node dissection after neoadjuvant ICB (nivolumab or nivolumab + ipilimumab) in HNSCC showed no tumour in 7 of 15 patients (47%) with an increase in lymph nodal SUV_max_ of 6 or more, and as much as 14 of 15 (93%) with a nodal SUV_max_ increase of 3 or more. However, they performed the second [^18^F]FDG-PET scan at a relatively early time point: median 14 days after ICB initiation, compared to 24 in our study [6]. Cervical lymph nodal pseudoprogression puts patients at risk of unjustified expansion of the cervical dissection, as was the case in one patient. Using a more tumour-specific radiotracer like 3′-deoxy-3′-[^18^F]fluorothymidine (FLT, a proliferation tracer) may help distinguish between true- and pseudoprogression and has previously been proven an early indicator of a favourable outcome after (chemo)radiotherapy in HNSCC [24, 25]. A small pilot study in stage IV melanoma patients treated with pembrolizumab (anti-PD-1) suggests FLT-PET-based response assessment in week 6 accurately predicts RECIST-based response in week 12, but its utility as a biomarker to separate pseudo- from truly progressive disease in ICB for HNSCC is unknown.

In conclusion, our data suggest that [^18^F]FDG-PET-based, primary tumour volumetric metabolic response assessment may be an early and accurate surrogate biomarker to identify individual HNSCC patients with a clinically relevant pathological response to neoadjuvant nivolumab or nivolumab + ipilimumab. In addition, an MTV or TLG decrease seems a promising tool to identify individual patients who are very unlikely to develop a tumour relapse, irrespective of mixed responses or pseudoprogression in the cervical lymph nodes, and may therefore serve as an on-treatment surrogate biomarker to guide response-driven treatment adaptation in future trials.

## Electronic supplementary material

Below is the link to the electronic supplementary material.

Supplementary file1 **Visualization of the patient with metabolic evidence of response, but without sufficient tumour regression to be a pathological responder. a, **A patient with cT3N1 HNSCC of the retromolar trigone and soft palate had no primary tumour pathological response, yet still demonstrated 22% tumour regression (not shown) and a decrease in SUV_max_ (-22%), SUV_mean_ (-7%), MTV (-47%), and TLG (-51%). **b**, A level 2 lymph node metastasis of the same patient shows a decrease in SUV_max_ (-36%), SUV_mean_ (-24%), MTV (-99%) and TLG (-99%). Correlative pathology (not shown) revealed a major pathological response in the lymph node metastasis (PDF 1582 KB)

## Data Availability

The datasets analysed in the current report are available from the corresponding author upon scientifically sound request. Every request will be reviewed by the Institutional Medical Ethics Committee of The Netherlands Cancer Institute (METC NKI) and the applying researcher will be required to sign a data access agreement with the NKI after approval.

## References

[1] Ferris RL, Blumenschein GJ, Fayette J, Guigay J, Colevas AD, Licitra L (2016). Nivolumab for recurrent squamous-cell carcinoma of the head and neck. N Engl J Med.

[2] Burtness B, Harrington KJ, Greil R, Soulières D, Tahara M, de Castro G, Jr. et al. Pembrolizumab alone or with chemotherapy versus cetuximab with chemotherapy for recurrent or metastatic squamous cell carcinoma of the head and neck (KEYNOTE-048): a randomised, open-label, phase 3 study. Lancet. 10.1016/S0140-6736(19)32591-7. 10.1016/S0140-6736(19)32591-7 31679945

[3] Blank CU, Rozeman EA, Fanchi LF, Sikorska K, van de Wiel B, Kvistborg P (2018). Neoadjuvant versus adjuvant ipilimumab plus nivolumab in macroscopic stage III melanoma. Nat Med.

[4] Chalabi M, Fanchi LF, Dijkstra KK, Van den Berg JG, Aalbers AG, Sikorska K (2020). Neoadjuvant immunotherapy leads to pathological responses in MMR-proficient and MMR-deficient early-stage colon cancers. Nat Med.

[5] van Dijk N, Gil-Jimenez A, Silina K, Hendricksen K, Smit LA, de Feijter JM (2020). Preoperative ipilimumab plus nivolumab in locoregionally advanced urothelial cancer: the NABUCCO trial. Nat Med.

[6] Schoenfeld JD, Hanna GJ, Jo VY, Rawal B, Chen Y-H, Catalano PS (2020). Neoadjuvant nivolumab or nivolumab plus ipilimumab in untreated oral cavity squamous cell carcinoma: a phase 2 open-label randomized clinical trial. JAMA Oncol.

[7] Vos JL, Elbers JBW, Krijgsman O, Traets JJH, Qiao X, van der Leun AM et al. Neoadjuvant immunotherapy with nivolumab and ipilimumab induces major pathological responses in patients with head and neck squamous cell carcinoma. Accepted, Nature Communications, 2021. 10.1038/s41467-021-26472-9 PMC8695578 34937871

[8] Rathod S, Livergant J, Klein J, Witterick I, Ringash J (2015). A systematic review of quality of life in head and neck cancer treated with surgery with or without adjuvant treatment. Oral Oncol.

[9] Eisenhauer EA, Therasse P, Bogaerts J, Schwartz LH, Sargent D, Ford R (2009). New response evaluation criteria in solid tumours: revised RECIST guideline (version 1.1). Eur J Cancer (Oxford, England: 1990).

[10] Forde PM, Chaft JE, Smith KN, Anagnostou V, Cottrell TR, Hellmann MD et al. Neoadjuvant PD-1 blockade in resectable lung cancer. 2018;378(21):1976–86. 10.1056/NEJMoa1716078. 10.1056/NEJMoa1716078 PMC6223617 29658848

[11] Wahl RL, Jacene H, Kasamon Y, Lodge MA (2009). From RECIST to PERCIST: evolving considerations for PET response criteria in solid tumors. J Nucl Med.

[12] Tao X, Li N, Wu N, He J, Ying J, Gao S (2020). The efficiency of (18)F-FDG PET-CT for predicting the major pathologic response to the neoadjuvant PD-1 blockade in resectable non-small cell lung cancer. Eur J Nucl Med Mol Imaging.

[13] Aide N, Hicks RJ, Le Tourneau C, Lheureux S, Fanti S, Lopci E (2019). FDG PET/CT for assessing tumour response to immunotherapy. Eur J Nucl Med Mol Imaging.

[14] Tetzlaff MT, Messina JL, Stein JE, Xu X, Amaria RN, Blank CU (2018). Pathological assessment of resection specimens after neoadjuvant therapy for metastatic melanoma. Ann Oncol.

[15] Young H, Baum R, Cremerius U, Herholz K, Hoekstra O, Lammertsma AA (1999). Measurement of clinical and subclinical tumour response using [18F]-fluorodeoxyglucose and positron emission tomography: review and 1999 EORTC recommendations. European Organization for Research and Treatment of Cancer (EORTC) PET Study Group. Eur J Cancer (Oxford, England : 1990).

[16] Uppaluri R, Campbell KM, Egloff AM, Zolkind P, Skidmore ZL, Nussenbaum B (2020). Neoadjuvant and adjuvant pembrolizumab in resectable locally advanced, human papillomavirus–unrelated head and neck cancer: a multicenter, phase II trial. Clin Cancer Res.

[17] Oliva M, Spreafico A, Taberna M, Alemany L, Coburn B, Mesia R (2019). Immune biomarkers of response to immune-checkpoint inhibitors in head and neck squamous cell carcinoma. Ann Oncol.

[18] Seymour L, Bogaerts J, Perrone A, Ford R, Schwartz LH, Mandrekar S (2017). iRECIST: guidelines for response criteria for use in trials testing immunotherapeutics. Lancet Oncol.

[19] Goldfarb L, Duchemann B, Chouahnia K, Zelek L, Soussan M (2019). Monitoring anti-PD-1-based immunotherapy in non-small cell lung cancer with FDG PET: introduction of iPERCIST. EJNMMI Res.

[20] Kaira K, Higuchi T, Naruse I, Arisaka Y, Tokue A, Altan B (2018). Metabolic activity by (18)F-FDG-PET/CT is predictive of early response after nivolumab in previously treated NSCLC. Eur J Nucl Med Mol Imaging.

[21] Umeda Y, Morikawa M, Anzai M, Ameshima S, Kadowaki M, Waseda Y (2020). Predictive value of integrated ^18^F-FDG PET/MRI in the early response to nivolumab in patients with previously treated non-small cell lung cancer. J Immunother Cancer.

[22] Blot WJ, McLaughlin JK, Winn DM, Austin DF, Greenberg RS, Preston-Martin S (1988). Smoking and drinking in relation to oral and pharyngeal cancer. Cancer Res.

[23] Johnson S, McDonald JT, Corsten MJ (2008). Socioeconomic factors in head and neck cancer. J Otolaryngol Head Neck Surg.

[24] Hoeben BAW, Troost EGC, Span PN, van Herpen CML, Bussink J, Oyen WJG (2013). 18F-FLT PET during radiotherapy or chemoradiotherapy in head and neck squamous cell carcinoma is an early predictor of outcome. J Nucl Med.

[25] Menda Y, Boles Ponto LL, Dornfeld KJ, Tewson TJ, Watkins GL, Schultz MK (2009). Kinetic analysis of 3′-deoxy-3′-(18)F-fluorothymidine ((18)F-FLT) in head and neck cancer patients before and early after initiation of chemoradiation therapy. J Nucl Med.

