# Supplementary figures and images for: [18F]FDG-PET accurately identifies pathological response early upon neoadjuvant immune checkpoint blockade in head and neck squamous cell carcinoma
Source: Eur J Nucl Med Mol Imaging. 2021 Dec 27;49(6):2010–22. doi: 10.1007/s00259-021-05610-x (PMC9016016; doi:10.1007/s00259-021-05610-x)

a.

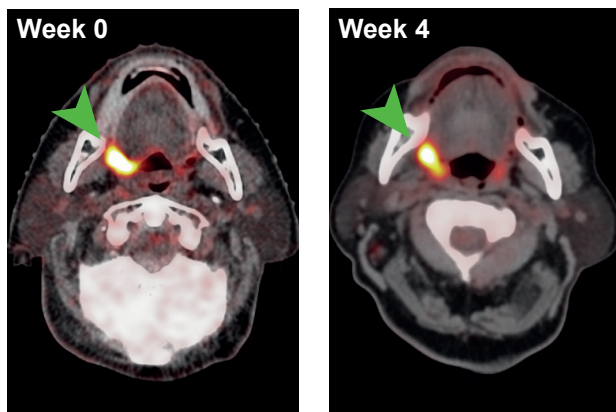

b.

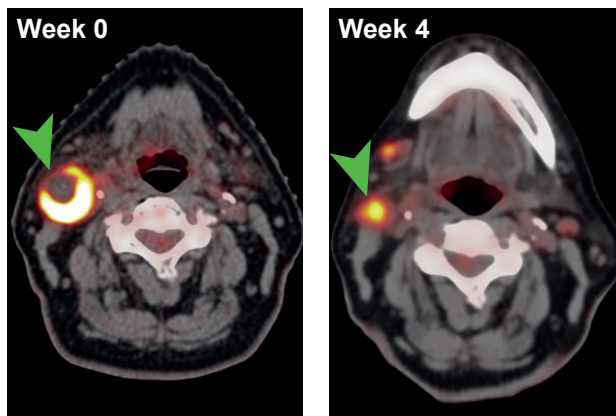

Supplement: Supplementary file 1 — Supplementary file1 Visualization of the patient with metabolic evidence of response, but without sufficient tumour regression to be a pathological responder. a, A patient with cT3N1 HNSCC of the retromolar trigone and soft palate had no primary tumour pathological response, yet still demonstrated 22% tumour regression (not shown) and a decrease in SUVmax (-22%), SUVmean (-7%), MTV (-47%), and TLG (-51%). b, A level 2 lymph node metastasis of the same patient shows a decrease in SUVmax (-36%), SUVmean (-24%), MTV (-99%) and TLG (-99%). Correlative pathology (not shown) revealed a major pathological response in the lymph node metastasis (PDF 1582 KB) [file 259_2021_5610_MOESM1_ESM.pdf]
